# Supplementary material for: Proteogenomics decodes the evolution of human ipsilateral breast cancer
Source: Commun Biol. 2023 Feb 2;6:139. doi: 10.1038/s42003-023-04526-6 (PMC9894938; doi:10.1038/s42003-023-04526-6)
Supplement: Supplementary file 3 — Description of Additional Supplementary Files [file 42003_2023_4526_MOESM3_ESM.pdf]

File Name: Supplementary Data 1
Description: Clinical and histopathological characteristics of patient samples included in this study

File Name: Supplementary Data 2
Description: Clinical and histopathological characteristics of primary tumors with no recurrence within 10 years

File Name: Supplementary Data 3
Description: Comparison between primary tumor cohorts

File Name: Supplementary Data 4
Description: Source data underlying main figures.
